# 2D weak anti-localization in thin films of the topological semimetal Pd$_{3}$Bi$_{2}$S$_{2}$

**DOI:** 10.1038/s41598-021-91930-9

**Published:** 2021-06-16

**Authors:** Shama, R. K. Gopal, Goutam Sheet, Yogesh Singh

**Affiliations:** Department of Physical Sciences, Indian Institute of Science Education and Research, Knowledge city, Sector 81, SAS Nagar, Manauli, Mohali, Punjab PO 140306 India

**Keywords:** Condensed-matter physics, Topological matter, Topological insulators

## Abstract

Pd$_{3}$Bi$_{2}$S$_{2}$ (PBS) is a recently proposed topological semimetal candidate. However, evidence for topological surface states have not yet been revealed in transport measurements due to the large mobility of bulk carriers. We report the growth and magneto-transport studies of PBS thin films where the mobility of the bulk carriers is reduced by two orders of magnitude, revealing for the first time, contributions from the 2-dimensional (2D) topological surface states in the observation of the 2D weak anti-localization (WAL) effect in magnetic field and angle dependent conductivity measurements. The magnetotransport data is analysed within the 2D Hikami-Larkin-Nagaoka (HLN) theory. The analysis suggests that multiple conduction channels contribute to the transport. It is also found that the temperature dependence of the dephasing length can’t be explained only by electron-electron scattering and that electron-phonon scattering also contributes to the phase relaxation mechanism in PBS films.

## Introduction

Topological materials such as topological insulators, topological semimetals, etc. have sparked worldwide interest owing to their novel physical properties and potential applications in the field of spintronics^1–5^. Topological Insulators (TI) have a bulk band gap with conducting edge or surface states protected by time-reversal symmetry (TRS)^1,2^. Topological semimetals (TSM) can be classified into Dirac semimetals and Weyl semimetals^3–5^. In a Dirac semimetal, conduction and valence band touch each other linearly at discrete points in the Brillouin zone called Dirac nodes which are protected by time-reversal symmetry (TRS) and Inversion symmetry (IS)^5,6^. On breaking either TRS or IS, this Dirac node splits into two Weyl nodes with opposite chiral charge, forming a Weyl semimetal^5^. The quasiparticles in Weyl and Dirac semimetals have two and four-fold degeneracy. Among the TSM candidates, Cd$_{3}$As$_{2}$ and Na$_{3}$Bi were first theoretically predicted and experimentally verified to host Dirac fermions^7–10^, whereas Weyl fermion were identified in the $TX (T =$ Ta, Nb; $X =$ As, P) family of materials^11–13^. The topological semimetals exhibit unusual phenomena such as extremely large magneto-resistance, chiral anomaly induced negative MR, high mobility, and anomalous Hall effect^14–17^.

Recently, based on certain space group symmetries, Bradlyn.*et al* have theoretically predicted the existence of exotic fermions (beyond Dirac, Weyl, Majorana) having three, six, and eightfold degenerate band crossing in various materials^18^. Pd$_{3}$Bi$_{2}$S$_{2}$ with space group 199 is one such material proposed to host exotic fermions having three-fold degenerate band crossing just 0.1 eV away from the Fermi level^18^. In previous studies, the magneto-transport has been reported on polycrystalline, single-crystalline, and nanoparticle samples of Pd$_{3}$Bi$_{2}$S$_{2}$^19–21^. The magneto-transport study on single-crystal indeed revealed interesting features like a large non-saturating magneto-resistance, which was ascribed to the high mobility of the charge carriers^21^.

To reveal contributions of the topological surface states it is essential to reduce the mobility of the bulk charge carriers. Additionally, reduction of system dimensionality from three dimensional (3D) to two dimensional (2D), is expected to lead to the emergence of several quantum phenomena at low temperatures, including weak localization (WL) or weak anti-localization (WAL), quantum interference (QI)^22–25^, and universal conductance fluctuations (UCF)^26^. The constructive or destructive interference of electrons moving through time reversed paths gives rise to negative or positive correction to conductivity, which is known as weak localization or anti-localization (WL/WAL)^22–25^. These effects can be experimentally verified using electron transport such as magneto-resistance (MR), Hall effect, etc. WAL is observed in topological materials, as an important consequence of spin momentum locking, resulting in a relative $\pi$ Berry phase acquired by electrons executing time-reversed paths^27,28^.

In this work, we report the first growth and characterization of Pd$_{3}$Bi$_{2}$S$_{2}$ (PBS) thin films using pulse laser deposition technique (PLD) and magneto-transport properties of PBS films prepared under different post annealing conditions. The mobility of the PBS films is found to be two orders of magnitude smaller than reported for bulk crystals. This reduced mobility of bulk carriers helps reveal the signature of topological surface states in quantum corrections to the angle-dependent magneto-transport. In the magnetic field dependent conductivity at low temperature, we have found a sharp cusp around $B = 0$ when the magnetic field is perpendicular to the film, revealing the presence of weak anti-localization. Applying the HLN theory, we have extracted the dephasing length (L$_{\phi }$) and the parameter $\alpha$ which depends on the number of conducting channels^27,29–31^. Our analysis suggests that both topologically non-trivial as well as trivial conduction channels contribute to the transport. Additionally we find that the temperature dependence of L$_{\phi }$ can not be described only by an electron-electron dephasing mechanism^32^. This indicates the existence of some other phase relaxation mechanism such as electron-phonon scattering in Pd$_{3}$Bi$_{2}$S$_{2}$ films^33–35^.

**Figure 1:**
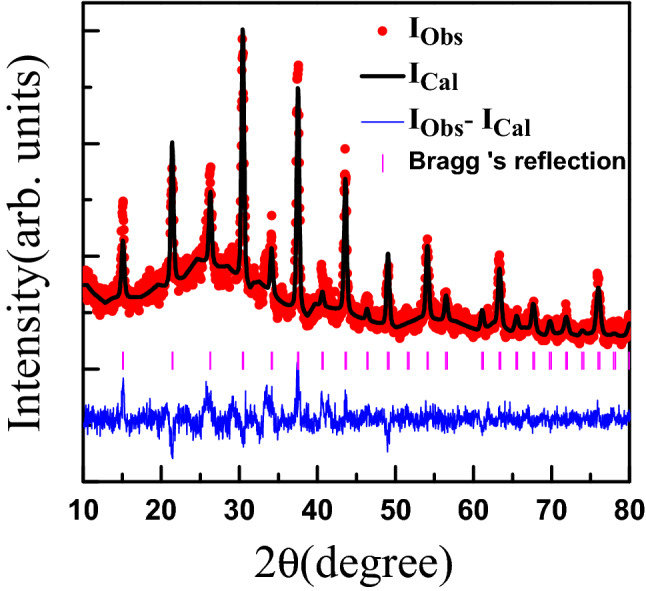
X-ray diffraction data (solid symbols) for as-grown (S0) Pd$_{3}$Bi$_{2}$S$_{2}$ film and it’s Rietveld refinement (solid curves through the data). The positions of the expected Bragg peaks are given as the vertical bars. The diference between the data and the refined pattern is shown at the bottom.

**Figure 2:**
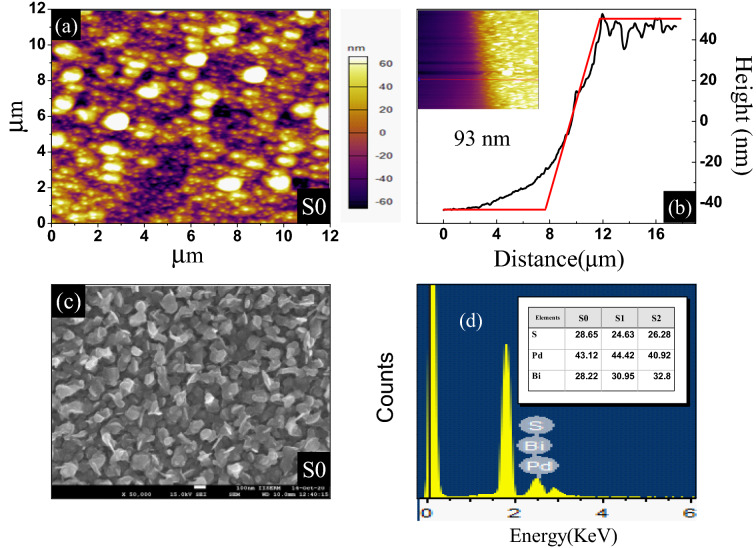
(**a**) Atomic Force Microscope topography image of the surface of the S0 PBS thin film. **(b**) The height profile across the S0 film with a thickness $\approx 93$ nm. (**c**) An SEM image of the surface of the S0 film. (**d**) Results of the EDS spectroscopy on S0 showing the presence of Pd, Bi, and S in stoichiometric amounts in the film.

## Experimental methods

Pd$_{3}$Bi$_{2}$S$_{2}$ thin films were grown on a Si(111) substrate using pulsed laser deposition ( KrF excimer , $\lambda$ = 248 nm) in Argon atmosphere. A polycrystalline target was prepared by the solid state reaction of stoichiometric amounts of high purity starting materials in a vacuum sealed quartz tube. The laser ablation was performed on the polycrystalline target under the growth conditions of 1.8 J/cm$^{2}$ laser fluence at low repetition rate of 1 Hz^36^. The as-grown PBS thin-films (S0) were post annealed for 30 min in Argon atmosphere at $260~^{\circ }$C (S1) and $300~^{\circ }$C (S2). An x-ray diffractometer (Bruker D8 Advance system with Cu-K$\alpha$ radiation) was used to determine the crystallographic phase of PBS thin-films. The stoichiometry of PBS thin films was confirmed using energy dispersive spectroscopy in a scanning electron microscope from JEOL. The surface morphology and average film thickness were measured using an atomic force microscope (AFM) from Asylum Research (model:MFP3D). The longitudinal and Hall resistances were measured in a Quantum Design Physical Property Measurement System (PPMS-ECII) equipped with a 9 T magnet.

## Results and discussion

Figure 1 shows the x-ray diffraction (XRD) pattern recorded at $T= 300$ K for the as-grown PBS thin film S0. The XRD pattern confirmed that our thin film crystallizes in the expected cubic crystal structure with space group I2$_{1}$3. A Rietveld refinement of the XRD pattern gave the lattice parameters $a = b = c = 8.47$ Å, which are in agreement with values reported previously for bulk PBS^19–21^. Figure 2a shows the atomic force microscope (AFM) topography image of the S0 thin film. This image indicates the polycrystalline granular growth of the film. We also determined the thickness of the film to be 93 nm as shown in Fig. 2b. Figure 2c shows the SEM image of S0. From the AFM and SEM images, it is concluded that PBS thin films are polycrystalline in nature. Figure 2d shows energy dispersive X-ray spectroscopy data for S0, where peaks of Bismuth, Palladium, and Sulfur are observed. The atomic ratio of these elements, shown in the Table in the inset, confirms the stoichiometry of the thin films. Therefore we conclude that we have successfully grown the first polycrystalline thin films of Pd$_{3}$Bi$_{2}$S$_{2}$. The AFM and SEM results for the S1 and S2 films, which lead to the same conclusion as for S0, are given in the Supplementary materials.

**Figure 3:**
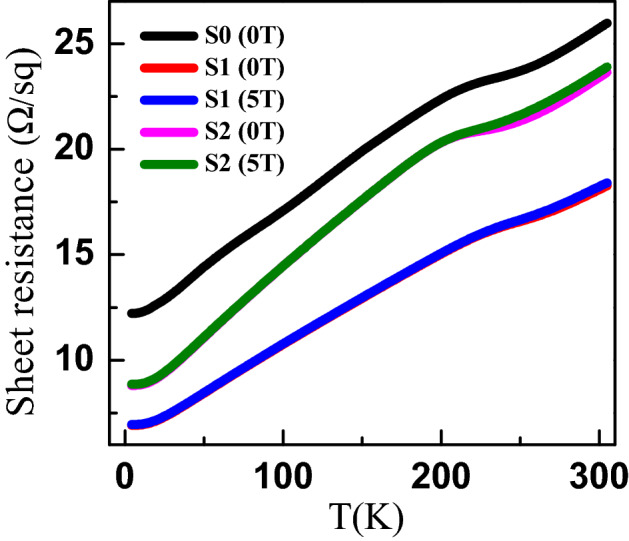
Sheet resistance vs temperature at $B = 0, 5$ T showing the metallic behaviour of S0–S2 films.

Figure 3 shows the variation of sheet resistance $R_S$ with temperature for PBS (S0–S2) thin films in magnetic fields $B = 0, 5$ T applied perpendicular to the film. The temperature dependence of $R_S$ reveals the metallic behavior of PBS thin fims with a large residual resistance $R_{o}$. This metallic behaviour is consistent with previous reports on single crystals^21^. We also observe a slope change in the sheet resistance close to 220 K seen in all films, which was not observed in bulk samples^19–21^. The origin of this slope change is not understood at present but we speculate that the connectivity between grains is changing with temperature, leading to a change in the conductivity of the films. To try to analyze how annealing affects the resistance of different films, we compare the residual resistivity ratio (RRR) and the residual resistivity $R_{o}$ of the as grown film S0 and the annealed films S1 and S2 in Table 1. From Fig. 3 and Table 1 we see that the RRR increases for the annealed films suggesting an improved quality arising most likely from the improved connectivity between grains. However, the $R_{o}$ varies non-monotonically, with a minimum value for the S1 films annealed at $260~^{\circ }$C, suggesting that the optimal annealing temperature for the PBS films may be $\sim 260^{\circ }$C.

**Figure 4:**
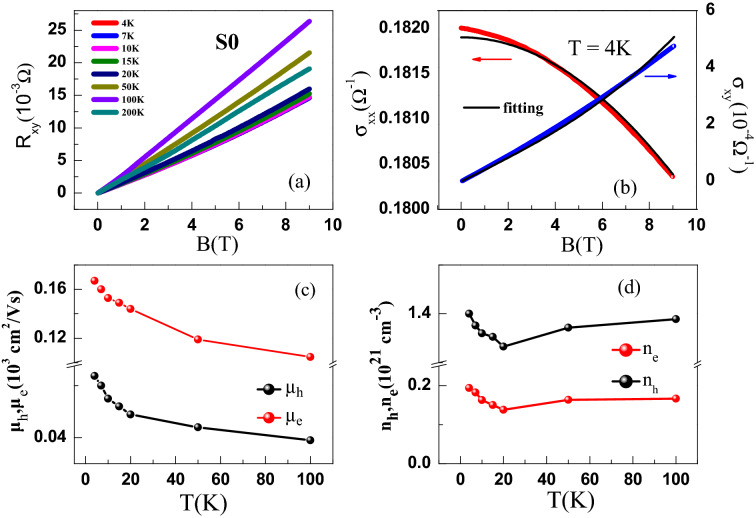
(**a**) Hall resistance (R$_{xy}$) vs magnetic field (*B*) at various temperatures for S0 thin film of PBS. (**b**) Variation of Hall conductance ($\sigma _{xy}$) and longitudinal conductance ($\sigma _{xx}$) as a function of *B* at $T = 4$ K. The solid curves through the data are the global fitting of the $\sigma _{xy}$ and $\sigma _{xx}$ by a two-band model (see text for details). (**c**) Temperature dependence of mobility and (**d**) carrier density of electron and hole carriers in S0. The solid curves through the data in (**c**) and (**d**) are guides to the eye.

**Table 1:** Parameters for Pd$_{3}$Bi$_{2}$S$_{2}$ thin films. $T_{\mathrm{ann}}$ is the post-annealing temperature, $R_o$ is the sheet resistance at the lowest temperature, RRR is the residual resistivity ratio, $n_e(n_h)$ is the electron(hole) carrier density, $\mu _e$($\mu _h$) is the electron(hole) mobility, L$_\phi$ is the phase coherence length, and $\alpha$ is a parameter in the HLN theory which depends on the number of cunduction channels.

	S0	S1	S2
T_ann_ ($^{\circ }$C)	Not annealed	260	300
$R_{o}$($\Omega$)	12.2	6.91	8.81
RRR	2.1	2.6	2.7
n$_e$/n$_h$($\times$ 10$^{21}$/cm$^{3}$)	0.19/1.4	0.21/1.6	0.2/1.53
$\mu$ $_{e}$/$\mu$ $_{h}$($\times$ 10$^{2}$cm$^{2}$/Vs)	1.7/0.64	1.5/0.6	1.6/0.61
L$_{\phi }$ (nm)	206	239	218
$\alpha$	0.28	0.26	0.29

**Figure 5:**
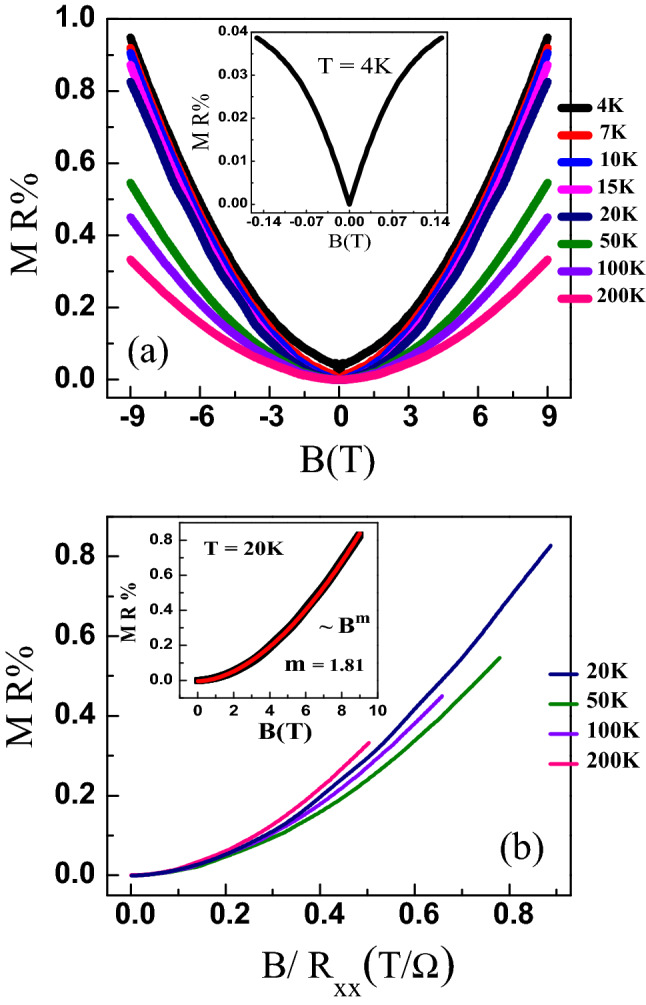
(**a**) The percentage magneto-resistance MR% vs magnetic field *B* at various temperatures for S0 film. The inset shows the data at low magnetic fields at $T= 4$K showing the WAL effect. (**b**) Violation of Kohler’s rule indicating the presence of multiple scattering mechanism. The inset shows the power-law $B^m$ fitting of the MR data at $T= 20$ K with $m = 1.81$.

Figure 4a shows the variation of Hall resistance (R$_{xy}$) with magnetic field (B) at various temperatures for S0 films. At high temperature, Hall resistance remains linear and positive with magnetic field for S0 as can be seen from Fig. 4a. At lower temperatures the Hall resistance becomes non-linear. Results for S1 and S2 are qualitatively similar and can be found in the Supplementary material. A non-linear Hall resistance implies the presence of more than one type of charge carriers in PBS. To extract the carrier concentration and mobility, we fit simultaneously and globally, the Hall conductance $\sigma _{xy}$ and the longitudinal conductance $\sigma _{xx}$ for all films to a semi-classical two-band model given by the expression^37,38^

1 $$\begin{aligned} \sigma _{xy}= & {} {eB}\left[ \dfrac{n_{h}\mu _{h}^{2}}{1+\left( {\mu _{h}B}\right) ^{2}}-\dfrac{n_{e}\mu _{e}^{2}}{1+\left( {\mu _{e}B}\right) ^{2}}\right] \end{aligned}$$

2 $$\begin{aligned} \sigma _{xx}= & {} e\left[ \dfrac{n_{h}\mu _{h}}{1+\left( {\mu _{h}B}\right) ^{2}}+\dfrac{n_{e}\mu _{e}}{1+\left( {\mu _{e}B}\right) ^{2}}\right] \end{aligned}$$

where *e* is the charge of an electron and *B* is the magnetic field. The *n* and $\mu$ are the carrier density and mobility, respectively. The subscript e, h denotes electrons and holes, respectively. The fit is shown as the solid curve through the $\sigma _{xx}$ and $\sigma _{xy}$ data for S0 in Fig. 4b and the parameters extracted from the fit to the data for all the films are given in Table 1. Figure 4c and d show the temperature dependence of mobility and carrier density for S0. The behaviour for S1 and S2 were found to be similar. The electron (hole) carrier density is estimated to be of order 10$^{20}$(10$^{21}$) cm$^{-3}$, which are similar to values reported previously in PBS single crystals. However, the mobility for the PBS films comes out to be of the order 10$^{2}$ cm$^{2}$/Vs which is smaller than reported in single crystals^21^. The reduced mobility may be due to a larger amount of disorder in the films compared to the single crystals as evidenced by the large residual resistance values. Additionally, the polycrystalline nature of the films may also lead to reduced mobility compared to crystals due to grain boundary scattering. From the extracted parameters we can rule out the possibility of charge carrier compensation as *p* is an order of magnitude larger than *n* indicating that holes are the dominant charge carriers in our PBS films.

An alternate procedure of fitting where $\sigma _{xx}$ and $\sigma _{xy}$ are fit separately using the respective expressions in Eqs. (1) and (2) can also be used instead of the simultaneous and global fit we have performed. Results of such a fit and parameters obtained from such a fit are included in the Supplementary materials.

**Figure 6:**
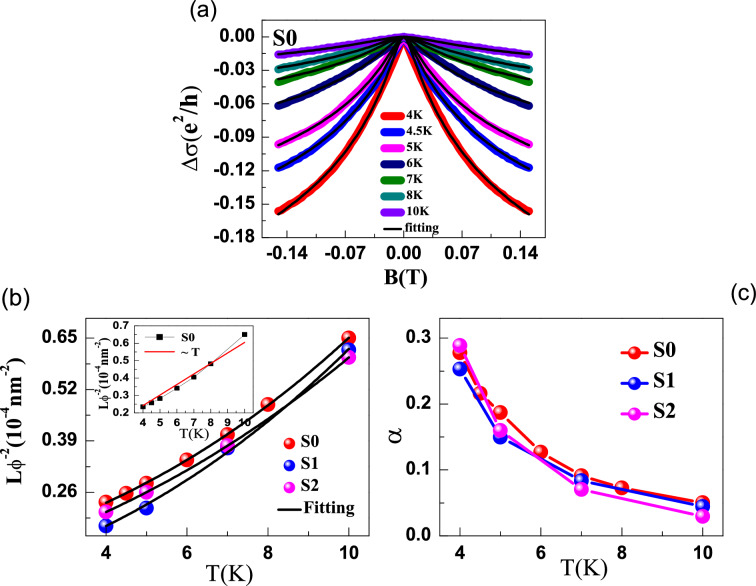
(**a**) The magneto-conductance ($\triangle \sigma$) versus field *B* at various temperatures for as-grown film S0. Solid curves through the data are fits to the HLN equation. (**b**) Variation of L$_{\phi }$ as a function of temperature, revealing the contribution of different scattering mechanisms. Inset shows the failure of a linear in *T* fitting. (**c**) Temperature dependence of $\alpha$.

Figure 5a shows the percentage magnetoresistance MR% for S0 at various temperatures where

3 $$\begin{aligned} {\mathrm{MR}}\% = \dfrac{R(B) - R(0)}{R(0)} \times 100~. \end{aligned}$$

Here *R*(*B*) is the sheet resistance in a magnetic field *B*. The inset in Fig. 5a shows the MR data at low fields highlighting the cusp-like behaviour at low magnetic fields. This cusp-like behavior under low field is a signature of weak anti-localization (WAL)^22–25^. The low value of the magneto-resistance in our films compared to the previous report on single crystals^21^ is mainly attributed to the lower carrier mobility in PBS thin films, as observed from the Hall data. According to Kohler’s rule, for materials with one dominant scattering mechanism, the MR can be represented as a universal function of the quantity $B\tau$, where $\tau$ is the time between scattering events of conduction electrons which is inversely proportional to the zero field resistivity $\rho$ $_{o}$^39^. We therefore expect that the MR is a universal function of the ratio $B/\rho _o$. Figure 5b shows the MR data for the S0 film plotted as a function of B/$R_{xx}$. If Kohler’s rule was obeyed, the MR vs B/$R_{xx}$ data would collapse onto a single curve. Figure 5b shows that the Kohler’s rule is violated for PBS films. This indicates the presence of more than one dominating scattering mechanism.

We next present the WAL analysis. However, the analysis depends on the dimensionality of the system. For thin films, we must determine whether the mean free path is greater than the film’s thickness. In our case, we have calculated the mean free path using the 2D formula, $l_{e} = \dfrac{\hbar }{e}\sqrt{2\pi n_{e}}\mu _{e}$, where n$_{e}$ and $\mu _{e}$ are the 2D Hall carrier density and mobility. We estimate $l_{e} = 117$ nm for S0, which is larger than the film thickness (93 nm). For Quantum interference (QI) effects, the relevant length scale is the phase coherence length $L_{\phi } = \sqrt{D\tau }$, where D is the diffusion constant and $\tau$ is the phase coherence time. Also, the criterion for 2D nature of thin films is $L_\phi>$ thickness of film^40,41^. In our case, the value of L$_{\phi }$ is found to be greater than the thickness of thin-film (based on the analysis presented below), indicating the 2D nature of our thin films.

Figure 6a shows the conductance at various temperatures in the low magnetic field range $|B| \le 0.15$ T for S0. The two dimensional conductance was found using $\triangle \sigma = \sigma \mathrm{(B)}-\sigma \mathrm{(0)}$ where $\sigma \mathrm{(B)} = {(\mathrm{L}/\mathrm{W})}$ (1/R$_{xx}$), and L and W are the length and width of film respectively. For two-dimensional systems, Hikami-Larkin-Nagaoka (HLN) equation can be used to model the effect of localization^23,24^. This theory involves the contribution of quantum effects from three different mechanisms, namely, spin-orbit coupling (SOC), elastic scattering, and electron phase coherence. In the limit of high SOC, Hikami-Larkin-Nagaoka (HLN) can be written as

4 $$\begin{aligned} \triangle \sigma \mathrm{(B)}= -\alpha \dfrac{e^{2}}{\pi h}\left[ \psi \left( \dfrac{1}{2}+\dfrac{B_{\phi }}{B} \right) - ln \left( \dfrac{B_{\phi }}{B}\right) \right] \end{aligned}$$

where $\psi$ is the digamma function, e is the electron charge, h is the Planck constant, B$_{\phi }$ = $\hbar ^{2}$/(4eL$_{\phi }^{2}$) is the characteristic field associated with phase coherence length L$_{\phi }$. The parameter $\alpha$ gives the number of conduction channels contributing to the transport. According to the HLN theory, $\alpha$ takes the value 1/2 for weak anti-localization (WAL). However, $\alpha$ has often been observed to deviate from 1/2 in the case when more than one conduction channels are available. In particular, $\alpha = 1/2$ only for a single topologically non-trivial conduction channel. If there are more than one topological channels, each will contribute $\alpha = 1/2$ and add up to give a combined $\alpha > 1/2$, whereas if there are multiple channels and some are topologically trivial while others are topological, it leads to $\alpha < 1/2$^27,29–31^.

We fit the experimental data by Eq. (4) with fitting parameters $\alpha$ and L$_{\phi }$. The temperature dependence of the obtained fitting parameters for all the films are shown in Fig. 6b,c. The extracted value of $\alpha = 0.28$ at $T= 4$ K is smaller than the theoretical value 0.5 expected for a single conduction channel. This indicates the presence of more than one conduction channel. For example, the presence of topologically trivial bands at the Fermi level could contribute to the conductivity in addition to the topological electrons. Figure 6c shows that $\alpha$ reduces from 0.28 at 4 K to 0.05 at 10 K, consistent with the trend previously observed for other topological materials like Cd$_3$As$_2$^27,29–31^.

**Figure 7:**
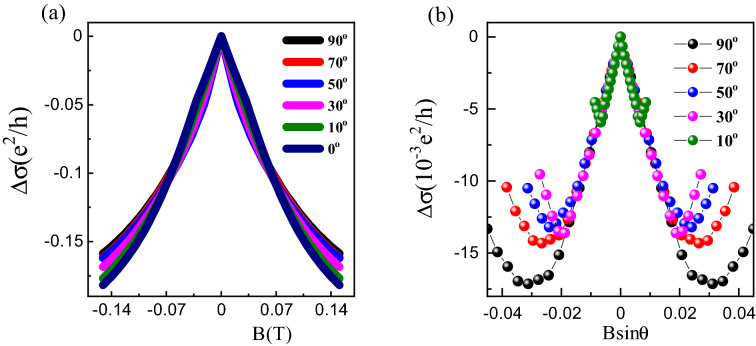
(**a**) The magneto-conductance ($\triangle$ $\sigma$) at $T = 2$ K measured with the field *B* applied at various angles $\theta$ to the current direction. (**b**) The 2D contribution to the magneto-conductance at various $\theta$ as a function of the perpendicular component of the applied magnetic field *B*Sin$\theta$.

Figure 6b shows the temperature dependence of phase coherence length L$_{\phi }$ plotted as $1/L_{\phi }^2$ versus *T* for as-grown and post annealed films. The L$_{\phi }$ decreases from 206 nm at 4 K to 124 nm at 10 K. The fact that L$_\phi$ comes out to be larger than the film thickness validates our use of 2D WAL analysis for our PBS films. The Nyquist theory, which considers electron-electron scaterring, predicts that L$_{\phi }$ $\propto$ T$^{-1/2}$ for 2D systems^32^. The inset of Fig. 6b shows that the L$_{\phi }$ data for PBS doesn’t follow the T$^{-1/2}$ dependence. This indicates that multiple scattering mechanisms with different temperature dependences could be involved in the dephasing of the electron’s phase in PBS. We try to analyze the temperature dependence of L$_\phi$ using the simple equation^33^

5 $$\begin{aligned} \dfrac{1}{L_{\phi }^{2}} =\dfrac{1}{L_{\phi o}^{2}} + A_{ee}T + B_{ep} T^p \end{aligned}$$

where $L_{\phi o}$ represents the zero temperature dephasing length, and A$_{ee}T$ and B$_{ep}$ T$^{p}$ represent the contributions from electron-electron and electron-phonon interactions, respectively. The value of *p* for electron-phonon interaction in 2D materials can vary between 2 to 3 depending on the effective dimensionality and disorder in the film^34,35,42,43^. We obtained an excellent fit of the temperature dependent L$_{\phi }$ data for all films by the Eq. (5) with $p \approx 2.4$ as shown by the solid curves through the data in Fig. 6b similar to values found on disordered films of GeSb$_2$Te$_4$^43^.

To further confirm that the WAL effect is coming from the 2D topological surface states and not the bulk bands, we investigated the angular dependence of the magneto-conductance. The conductance at $$T = 2$$ K as a function of magnetic field applied at various angles $$\theta $$ to the current, is shown in Fig. 7a. To reveal contribution from the 2D surface states we have subtracted the $$\theta = 0$$ data from those at finite $$\theta $$ and plotted the difference as a function of the perpendicular field component *B*Sin$$\theta $$ as shown in Fig. 7b. It is clear that at low values of the perpendicular field, before the upturn in the data due to contribution from bulk bands, all data measured at various $$\theta $$ collapse onto a common curve demonstrating the dominant contribution to the WAL from 2D topological surface states. Such scaling of tilt angle dependent conductivity data demonstrating 2D WAL from topological surface states has been observed previously in many topological materials like single crystals of Bi$$_2$$Se$$_{2.1}$$Te$$_{0.9}$$^44^, nano-flakes of Bi$$_2$$(Se$$_x$$Te$$_{1-x})_3$$^45^, low mobility crystals of LuPdBi^46^, few layer devices of ZrTe$$_5$$^47^, and disordered thin films of Bi$$_2$$Te$$_3$$^48^.

## Conclusion

We report the first synthesis of thin films (93 nm) of the novel topological material Pd$_3$Bi$_2$S$_2$ grown on Si (111) substrate by pulsed laser deposition. Longitudinal resistance measurements on PBS thin films indicate a disordered metallic system. This results in a mobility of bulk carriers which is two orders of magnitude reduced compared to single crystals. This suppresses the bulk contribution and allows the first detection of transport contribution from topological surface states through the observation of the 2D WAL effect. The WAL data measured for fields applied at different angles to the current direction, all scale with the perpendicular component of the field, confirming the contribution of 2D topological surface states to the WAL. These results are satisfactorily analysed in terms of Hikami-Larkin-Nagaoka theory. It was found that the coefficient $\alpha$ deviates from the value 0.5 expected for 2D systems with a single topological conduction channel. This indicates the contribution from additional (topological and trivial) conducting channels in the electron transport of PBS films. Dependence of the dephasing length L$_{\phi }$ on temperature is also anomalous. We found that this behaviour can be understood by including both the Nyquist electron-electron scattering as well as electron-phonon scattering as the phase relaxation mechanism in PBS films. The magneto-resistance data show deviations from Koehler’s rule suggesting the presence of multiple scattering mechanisms. These anomalous behaviours make Pd$_3$Bi$_2$S$_2$ an interesting system for further study in various morphologies.

## Supplementary information

Supplementary material 1.
