# Supplementary material for: 2D weak anti-localization in thin films of the topological semimetal Pd3Bi2S2
Source: Sci Rep. 2021 Jun 16;11:12618. doi: 10.1038/s41598-021-91930-9 (PMC8209139; doi:10.1038/s41598-021-91930-9)
Supplement: Supplementary file 1 — Supplementary material 1. [file 41598_2021_91930_MOESM1_ESM.pdf]

Supplementary Material for : 2D Weak Anti-localization in thin films of the Topological Semimetal $Pd_3Bi_2S_2$

Shama,[1] R. K. Gopal,[1] Goutam Sheet,[1] and Yogesh Singh[1,*]

[1]*Department of Physical Sciences, Indian Institute of Science Education and Research, Knowledge city, Sector 81, SAS Nagar, Manauli PO 140306, Mohali, Punjab, India*

This supplementary materials section presents atomic force microscopy (AFM), scanning electron microscopy (SEM), and transverse and longitudinal magneto-conductance results for the $Pd_3Bi_2S_2$ films annealed at 260°C (S1) and at 300°C (S2). Additionally, it includes results of separate fits to the Hall conductivity σ_{xy} and longitudinal conductivity σ_{xx} vs field of the as grown film S0 to contrast with the global and simultaneous fits to these two sets of data presented in the main paper.

I. CHARACTERISATION AND MAGNETO-TRANSPORT ON FILMS S1 AND S2

Figure 1 (a, b) shows the atomic force microscope (AFM) topography image of the S1 and S2 thin films. Figure 1 (c, d) shows the SEM image of S1 and S2. From the AFM and SEM images, it is concluded that PBS thin films are polycrystalline in nature.

Figure 2(a, b) shows the variation of Hall resistance (R_{xy}) with magnetic field (B) at various temperatures for S1 and S2 films. At high temperature, Hall resistance remains linear and positive with magnetic field. However, at lower temperatures the Hall resistance becomes non-linear. Consistent with results for S0 this suggests the presence of more than one type of charge carriers.

Figure 3(a, b) shows the conductance at various temperatures in the low magnetic field range $|B| \leq 0.15$ T for S1 and S2. The two dimensional conductance was found using $\triangle\sigma = \sigma$(B)-σ(0) where σ(B) = (L/W) (1/R_{xx}), and L and W are the length and width of the film respectively. The cusp-like behavior

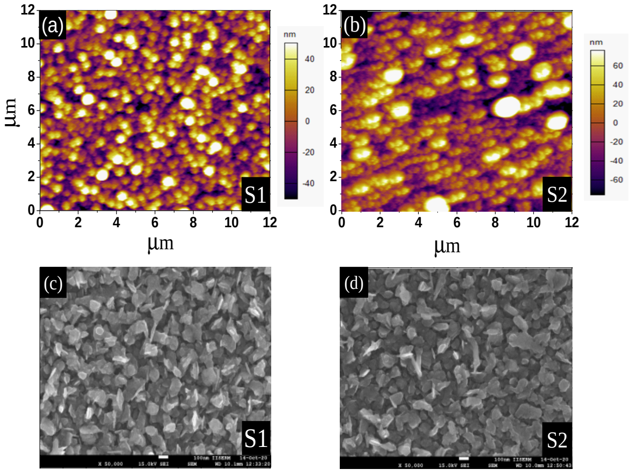

FIG. 1: The AFM topographic images (a, b) and the SEM topographic images (c, d) for S1 and S2 PBS films.

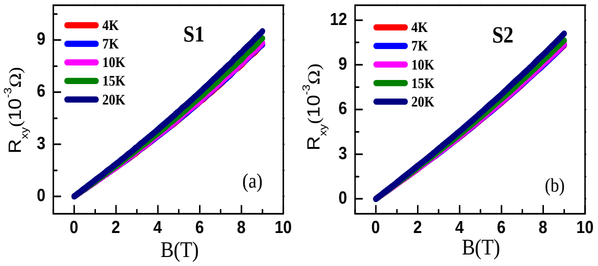

FIG. 2: The Hall resistance (R_{xy}) vs magnetic field (B) at various temperatures for (a) S1 and (b) S2 PBS films.

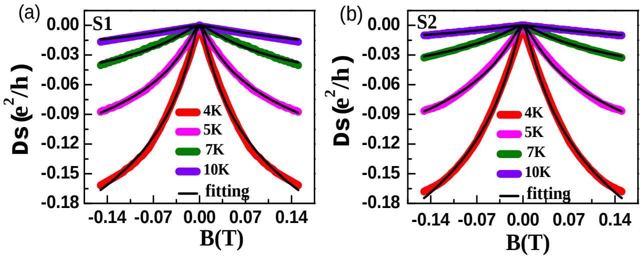

FIG. 3: The magneto-conductance ($\Delta\sigma$) versus field B at various temperatures for S1 (a) and S2 (b) films. The solid curves through the data are fits to the HLN equation.

under low field is a signature of weak anti-localization (WAL) For two-dimensional systems, Hikami-Larkin-Nagaoka (HLN) equation can be used to model the effect of localization.[1,2] This theory involves the contribution of quantum effects from three different mechanisms, namely, spin-orbit coupling (SOC), elastic scattering, and electron phase coherence. In the limit of high SOC, Hikami-Larkin-Nagaoka (HLN) can be written as

$$\triangle\,\sigma(\mathrm{B}) = -\alpha\frac{e^2}{\pi h}\left[\psi\left(\frac{1}{2}+\frac{B_\phi}{B}\right) - \ln\left(\frac{B_\phi}{B}\right)\right] \quad (1)$$

where ψ is the digamma function, e is the electron

charge, h is the Planck constant, $B_\phi = \hbar^2/(4eL_\phi^2)$ is the characteristic field associated with phase coherence length L_ϕ. The parameter α indicates the number of conduction channels contributing to the transport.[3–6]

We fit the experimental data by Eq. 1 with fitting parameters α and L_ϕ. The temperature dependence of the obtained fitting parameters for all the films was already shown in Fig. 6 of the main manuscript. The extracted value of $\alpha = 0.26(0.29)$ at $T = 4$ K for S1(S2) is smaller than the theoretical value 0.5 expected for a single topological conduction channel. This indicates the presence of both topological and trivial conduction channels. The value and the temperature dependence of α for S1 and S2 are consistent with the trend previously observed for other topological materials like Cd_3As_2.[3–6]

II. ALTERNATE FITTING OF σ_{xx} AND σ_{xy} FOR S0

In the main manuscript, we had performed a simultaneous and global fit to the Hall conductance σ_{xy} and the longitudinal conductance σ_{xx} for all films using a semi-classical two-band model given by the expressions

$$\sigma_{xy} = eB\left[\frac{n_h\mu_h^2}{1+(\mu_h B)^2} - \frac{n_e\mu_e^2}{1+(\mu_e B)^2}\right] \quad (2)$$

$$\sigma_{xx} = e\left[\frac{n_h\mu_h}{1+(\mu_h B)^2} + \frac{n_e\mu_e}{1+(\mu_e B)^2}\right] \quad (3)$$

where e is the charge of an electron and B is the magnetic field. The n and μ are the carrier density and mobility, respectively. The subscript e, h denotes electrons and holes, respectively.

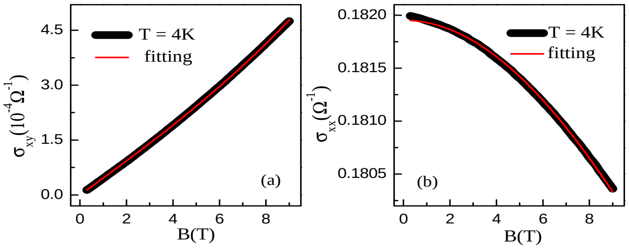

FIG. 4: The fitting of the transverse Hall σ_{xy} and the longitudinal σ_{xx} conductivity using a two band model.

Alternately, one may fit σ_{xx} and σ_{xy} separately using the respective expression given above. We have made such a fit which is shown as the solid curve through the σ_{xx} and σ_{xy} data for S0 in Fig. 4 and the parameters extracted from the fit are given in Table I.

* yogesh@iisermohali.ac.in

1 Hikami, S., Larkin, A. Nagaoka, Y., Prog. Theor. Phys. **63**, 707 (1980).
2 L. Fang, J. Im, W. DeGottardi, Y. Jia, A. Glatz, K. A. Matveev, W.-K. Kwok, G. W. Crabtree, M. G. Kanatzidis, Sci. Rep. **6**, 35313 (2016).
3 H.-Z. Lu and S.-Q. Shen, Phys. Rev. B **84**, 125138 (2011).
4 Z. Li, et al., Phys. Rev. B **91**, 041401 (2015).
5 B. Zhao, P. Cheng, H. Pan, S. Zhang, B. Wang, G. Wang, F. Xiu, F. Song, Sci. Rep. **6**, 22377 (2016).
6 H. Cao, C. Liu, J. Tian, Y. Xu, I. Miotkowski, M. Z. Hasan, and Y. P. Chen, arXiv:1409.3217.

TABLE I: Parameters obtained from fitting of σ_{xy} and σ_{xx} for the as-grown film S0 to Eqs.2 and 3, respectively. Here $n_e(n_h)$ is the electron(hole) carrier density and $\mu_e(\mu_h)$ is the electron(hole) mobility.

Fitting of	n_e(cm^{-3})	n_h(cm^{-3})	μ_e(cm^2/V s)	μ_h(cm^2/V s)
σ_{xx}	1.3×10^{20}	24.8×10^{20}	459	48
σ_{xy}	4.2×10^{20}	11.3×10^{20}	123	77
